# Adherence in Internet Interventions for Anxiety and Depression: Systematic Review

**DOI:** 10.2196/jmir.1194

**Published:** 2009-04-24

**Authors:** Helen Christensen, Kathleen M Griffiths, Louise Farrer

**Affiliations:** ^1^Centre for Mental Health ResearchThe Australian National UniversityCanberraAustralia

**Keywords:** Patient dropouts, depression, depressive disorder, major, anxiety disorders, Internet, mental health services, treatment outcome

## Abstract

**Background:**

Open access websites which deliver cognitive and behavioral interventions for anxiety and depression are characterised by poor adherence. We need to understand more about adherence in order to maximize the impact of Internet-based interventions on the disease burden associated with common mental disorders.

**Objective:**

The aims of this paper are to review briefly the adherence literature with respect to Internet interventions and to investigate the rates of dropout and compliance in randomized controlled trials of anxiety and depression Web studies.

**Methods:**

A systematic review of randomized controlled trials using Internet interventions for anxiety and depression was conducted, and data was collected on dropout and adherence, predictors of adherence, and reasons for dropout.

**Results:**

Relative to reported rates of dropout from open access sites, the present study found that the rates of attrition in randomized controlled trials were lower, ranging from approximately 1 - 50%. Predictors of adherence included disease severity, treatment length, and chronicity. Very few studies formally examined reasons for dropout, and most studies failed to use appropriate statistical techniques to analyze missing data.

**Conclusions:**

Dropout rates from randomized controlled trials of Web interventions are low relative to dropout from open access websites. The development of theoretical models of adherence is as important in the area of Internet intervention research as it is in the behavioral health literature. Disease-based factors in anxiety and depression need further investigation.

## Introduction

Web-based interventions are effective for a range of mental health disorders including depression, panic, post-traumatic stress disorder (PTSD), perceived stress in schizophrenia, stress, insomnia, and eating disorders [1]. While efficacy trials of Web interventions show good-to-excellent levels of adherence, open access websites have been associated with poor adherence and dropout, with substantial numbers of users not completing all Web pages and exiting websites before the full completion of an offered program [2,3]. For example, Farvolden [4] found that only 1% of participants completed a 12-week open access panic program, and Christensen and colleagues [5] reported that less than 1% of users completed all modules in an open access website for depression.

There is little reason to expect that the rates of adherence to websites offered as open access on the Web would be as strong as those reported for websites which are examined in the context of an efficacy trial. Open access websites provide information and Web content directly to community users at no, or minimal, cost. Data on adherence from these sites is based on the activity of spontaneous users who “visit” these sites, where many users will have no expectation that they will be offered “programs”. In contrast, data from efficacy trials of websites are based on responses from participants who are recruited to the trial on the basis of elevated symptoms; consent in advance of the trial; are provided with information about its parameters (nature and length of the program, etc); and are required to complete pre- and post-intervention surveys. Nevertheless, we need to know more about the basis of adherence and dropout, not least because there is evidence that greater exposure to website content is associated with increased benefit [6,7]. The Internet platform offers the opportunity to yield a rich source of objective data on engagement and dropout, and, consequently, has the potential to inform adherence research generally. High quality, objectively measurable information on treatment compliance can be obtained from logs of page views, resource downloads, time on site, and other indicators of treatment exposure. High volume Internet sites have the potential to investigate the effect of theory-driven modifications on adherence through the use of high throughput online randomized controlled trials (see, for example, Christensen and Mackinnon [8]).

The present paper has four aims: (1) to undertake a systematic review of the rates of adherence in randomized controlled trials (RCTs) of Internet interventions for anxiety and depression, with the aim of determining rates of attrition in order to confirm that Internet rates of attrition are lower in research trials than open access websites; (2) to collate data from these RCTs to identify predictors of dropout and adherence; (3) to examine the research studies for data on participant’s perceptions of adherence and dropout; and (4) to examine the type of analyses that were used to manage “missingness”, given that dropout from RCTs needs to be considered in every analysis of efficacy. To our knowledge, only two papers have reported rates of dropout from open access websites [4,5], but no systematic review of adherence or dropout from RCTs has been undertaken.

This paper begins by providing the context for these aims by defining adherence and dropout, briefly reviewing the research strategies used to investigate adherence in both Internet and non-Internet trials, and describing the evidence arising from these strategies. Research indicates that there are differences in the predictors of adherence for different health conditions [9]. Hence, we restrict our review to websites that target anxiety and depression. A brief discussion of approaches to the statistical analysis of dropout is also presented.

### Definitions

Most definitions of adherence are not well suited to the characteristics of e-interventions. For example, the World Health Organization (WHO) describes adherence as the “extent to which a person’s behavior [...] corresponds with agreed recommendations from a health care provider” [10]. This definition clearly does not transfer readily to the Web environment particularly with respect to interventions that are designed to be offered through open access sites, or to interventions that are predicated on self-help models. In the context of this paper, the term *adherence* refers to the extent to which individuals experience the content of the Internet intervention. The term *dropout* is used to describe an individual who fails to complete the research trial protocol associated with an Internet intervention, and thus does not complete trial assessments. These terms correspond reasonably closely to Eysenbach’s terms “non-usage dropout attrition” and “non-usage attrition”, which he applied to the uptake of Internet interventions. Dropout attrition refers to loss of participants from the trial [2]. Non-usage refers to participants’ lack of exposure to the website material. While it is perhaps simpler to use the terms usage and dropout attrition with respect to Web interventions, it is important also to “mainstream” Internet interventions—that is, to provide appraisals of them using terms appropriate to formal non-Internet based trials. For this reason, we use the terms adherence and dropout for the remainder of the article.

Clearly, dropout and treatment adherence refer to interrelated but conceptually distinct constructs. Individuals may drop out of a trial (fail to complete assessments) but have 100% treatment adherence. This occurs, for example, when users continue to undertake the prescribed program even though they have severed contact with the research or clinical team. Others may complete the protocol fully but adhere to the intervention less than 100% of the time. In this case, participants do not undertake the full Web program, although they may continue to complete all assessments.

**Figure 1:**
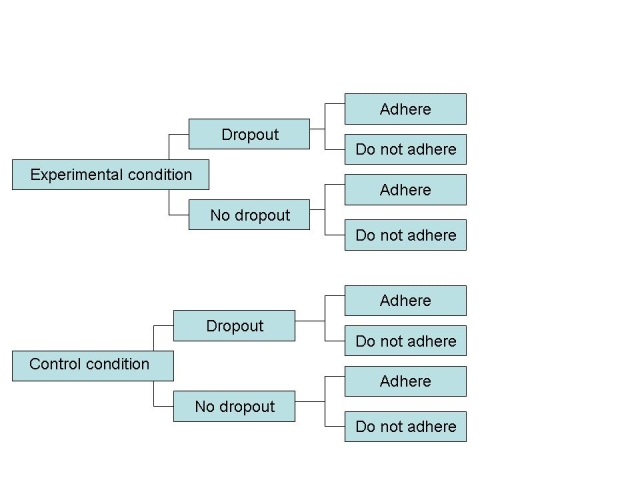
The relationship between dropout and adherencein a two-arm trial

Figure 1 outlines the potential range of outcomes of dropout and adherence in terms of a two-arm research trial. This represents a simplified analysis because there may be degrees or levels of adherence and dropout. Participants may complete an interim follow-up but not the final follow-up, or miss interim assessments but complete final assessments, and so on (ie, participants are classified as dropouts at various stages of the study).

The figure serves as a schematic to highlight important questions, such as how adherence is measured if participants drop out informally or formally by withdrawing their consent. Dropout increases progressively with program length [11], so direct comparisons of percentage dropout are not appropriate unless the length of the programs are roughly equivalent. Within the research trial context, dropouts have been subclassified as *no shows*, (those who do not proceed to the consent or treatment stage and do not complete assessments), *early dropouts* (those who drop out relatively early in a program and complete only one or very few assessments), or *late dropouts* [12]. The term *early completer* has been used to refer to those who benefit from the intervention but do not complete the protocol. In the Web context, these participants have been referred to “e-attainers” [8].

### Strategies to Studying Adherence

Three general approaches are undertaken to investigate adherence in both Web and non-Web environments. The first approach involves using correlational or regression analyses within trials to establish associations between adherence and various personality, demographic, and service delivery factors. Common variables investigated include aspects of service delivery, therapist factors, rewards and incentives, program duration, the nature of the medium of delivery, and personality factors, including expectations, self-determination, self-efficacy, or support from partners and friends (Davis and Addis [12], page 347). Disease-specific effects such as disease severity predict adherence, with a high level of emotional distress leading to early dropout [12]. Demographic variables, such as age, socioeconomic status, education, and marital status typically do not predict patient adherence across a range of health conditions [13]. For depression, the results from 14 epidemiological studies failed to indicate any clear predictors of adherence to medication regimes, although adverse side effects reduced adherence [14]. The amount of variance explained by these investigations is low. Moreover, these categories do not take into account the unique type and range of adherence variables associated with Internet delivery, and additional work is needed to investigate “computer factors” rather than therapist factors. With respect to e-health applications, one example of this correlational approach is a study of 82,000 users of an open access website for depression [15].

A second approach involves the use of post-test questionnaires to obtain retrospective analyses of people’s perceptions of trial participation, barriers to the use of the treatment, and other factors. An example of this approach is Ritterband’s follow-up interview which has been used to identify barriers to seeking out and using a paediatric website [16].

A third approach involves the experimental manipulation of variables believed to be causal in promoting adherence. A recent review summarized the effects of 38 systematic reviews of the effectiveness of adherence interventions across a range of disease conditions and intervention types published between 1990 and 2005 [9]. Interventions to improve adherence were classified into four types: technical solutions (such as simplifying doses); behavioral interventions; educational programs; and social support interventions. This review revealed that less frequent medication schedules (fewer but higher dose tablets) increased adherence (for most targeted disorders, with the exception of depression, see Yildiz et al [17]), as did behavioral interventions which provided reminders, used monitoring, or introduced rewards. Educational interventions were successful when patients were trained in cognitive problem solving or when they were taught motivational techniques. However, knowledge alone was not successful. Little evidence was found for the effectiveness of social support interventions. A number of studies investigated adherence to anxiety and depression interventions [9]. These interventions were complex, involving many components of collaborative care. Consequently, the specific components that were critical in improving adherence were difficult to identify [14], although collaborative care produced better adherence than educational interventions directed at the provider [10,18]. To date, in the e-health field, there has been little experimental manipulation of factors likely to increase adherence in e-health trials.

### Approaches Missing Data Arising From Dropout

Intention to treat analyses (ITT), where all participants in the trial are analyzed regardless of whether they drop out, is recommended for publication in most large-scale research studies, although the extent to which this approach is undertaken in RCTs of Internet trials is not known. ITT analyses take into account bias arising from selective attrition and hence are preferred over completer analyses, where only those completing the protocol are analyzed. Missing data for ITT approaches can be managed in a number of ways, including through the use of last observation carried forward (LOCF) imputation. However, more advanced methods which include the use of multiple imputation [19,20] and maximum-likelihood based methods [21], are more likely to yield valid outcomes. The use of ITT and methods to manage missing data is examined in the present review.

## Methods

### Study Selection

Relevant studies were identified using the methodology employed in our previous systematic reviews of RCTs of mental health Internet interventions [1,22]. The databases of PubMed, PsycInfo, and Cochrane Register Randomized Controlled Trials were searched using the key terms “Internet OR Web” together with search strategies designed to capture randomized controlled trials. Criteria for inclusion of a study in the current review were that it (1) involved a self-help website for a depressive or anxiety disorder; (2) tested the efficacy of a self-help psychoeducational or skills training intervention; (3) employed a randomized controlled trial design; and (4) incorporated a control group that was not subjected to an active treatment intervention. Only peer reviewed published articles were included in the analysis. Dissertations and published poster abstracts were excluded. Because our previous reviews collected information prior to 2007, we updated the search to include papers published before February 2009. For this update, a total of 1177 abstracts were retrieved from the searches conducted in PubMed, PsycInfo, and Cochrane Controlled Trials.  Of these, 1154 papers were excluded because they did not describe a self-help website for a depression or anxiety disorder, 2 were excluded because the intervention they described did not test the efficacy of a psychoeducational or skills training program, 3 were excluded because they did not employ a randomized controlled trial design, 7 were excluded because they used a control group that was subjected to an active treatment, and 2 were excluded because they were not published as a peer reviewed paper.  The remaining 9 studies met the criteria for inclusion in the review, and we added them to the original 14 studies.

### Coding of Study Characteristics

#### Sample Size

The number of participants in the study intervention was recorded.

#### Dropout

Dropout was defined as the number of individuals who failed to complete the research protocol. Typically, these figures were derived from the trial flow diagram. Thus, dropouts were those who failed to complete post-treatment or follow-up assessments once they had been accepted into the trial.

#### Adherence

Adherence was defined as an indicator of the extent to which individuals used the material on the website. Information on adherence was collected and reported with respect to logons, time on site, and number of modules attempted.

#### Predictors of Adherence or Dropout

We also recorded any reported association between a predictor (gender, severity) and any dropout or adherence measure.

#### Reason for Dropout

Any data on the reason for dropout was recorded.

#### Types of Statistical Analyses

Analyses were classified as either completer or intention to treat (ITT), with the method used to handle missing data noted.

## Results

There were 8 trials of depression interventions, 1 trial of a depression, anxiety, and stress intervention, 1 trial of a generalized anxiety disorder (GAD) intervention, 5 trials of panic disorder (PD) interventions, 4 trials of social phobia (SP) interventions, and 4 trials of Post Traumatic Stress Disorder (PTSD) interventions (Table 1).

**Table 1:** Summary of included randomized controlled trials of Internet interventions for anxiety and depression

	Sample size N = total I = Intervention C = Control	Dropout N = total I = Intervention C = Control	Adherence to treatment	Predictors of dropout/adherence	Self-reported reason for dropout?	Type of statistical analysis: ITT, NMAR, MAR, LOCF
**Depression**						
Andersson et al 2002 [23] (6 modules)	N = 117 I = 53 C = 64	Post-treatment: N = 66 (56.4%) I = 23 (43.4%) C = 43 (67.2%) 1 year: N = 96 (82.1%) I = 46 (86.8%) C = 50 (78.1%)	Not reported. Mean posting on discussion board = 8.7 (SD = 21.5)	Response rate higher in control group at post-treatment.	No formal measure described. Reported reasons: lack of time, programme too fast, lack of ideal environment to complete programme, programme is impersonal and too extensive.	Completers. Multiple regression
Andersson et al 2005 [24] (5 modules + discussion group)	N = 117 I = 57 C = 60^a^	Post-treatment: N = 85 (72.6%) I = 36 (63.1%) C = 49 (81.6%) 6 months: N = 71 (60.7%) I = 36 (63.1%) C = 35 (58.3%)^b^	Mean number of modules completed = 3.7 out of 5 (SD = 1.9) Total postings on the discussion board: I = 233 C = 842 (C > I, *P* < .05)	Lower withdrawal for control than treatment group participants at 3 months. (100% intervention completed; 71% Control group completed). No significant differences in depressive symptoms (BDI) or age, gender, educational level, place of living, or quality of life between dropouts and completers at 3 months.	No formal measure described. Main reported reason: treatment was too demanding.	ITT LOCF, ANOVA
Christensen et al 2004 [6] Griffiths et al 2004 [25] Mackinnon et al 2008 [26] (12 month follow-up) (5 modules)	N = 525 I(i) = 165 I(ii) = 182 C = 178 *I(i) = BluePages depression informationI(ii) = MoodGYM*^c^ *CBT*	Post-treatment: N = 435 (82.8%) I(i) = 140 (84.8%) I(ii) = 136 (74.7%) C = 159 (89.3%) 6 months: N = 352 (67%) I(i) = 115 (69.6%) I(ii) = 106 (58.2%) C = 131 (73.6%) 12 months: N = 325 (61.9%) I(i) = 107 (64.8%) I(ii) = 94 (51.6%) C = 124 (69.6%)	Mean BluePages visits = 4.49 (SD = 1.4) Mean MoodGYM exercises completed = 14.8 (SD = 9.7) (51%)	Greater dropout for MoodGYM (CBT) than BluePages (depression information) (*P* = .0001) Baseline depressive symptoms (CES-D) and knowledge of psychological treatments lower among dropouts (*P* < .01) Males more likely to be lost to follow-up at 12 months.	No	ITT LOCF
Clarke et al 2002 [27] (7 content chapters)	N = 299 I = 144 C = 155	4 weeks: N = 158 (52.8%) 8 weeks: N = 195 (65.2%) 16 weeks: N = 196 (65.6%) 32 weeks: N = 177 (59.2%)	Not reported. Mean logons: I = 2.6 (SD = 2.5; range 1-20)	Baseline depressive symptoms (CES-D) lower in those who completed at least one follow-up questionnaire (*P* < .05). Age, gender, recruitment group did not predict dropout.	No	ITT random effect regression analyses
Clarke et al 2005 [28] (7 content chapters)	N = 255 I(i) = 75 I(ii) = 80 C = 100 *I(i) = website + postcard remindersI(ii) = website + telephone reminders*	5 weeks: N = 164 (63.1%) I(i) = 36 (48%) I(ii) = 48 (60%) C = 77 (77%) 10 weeks: N = 173 (67.8%) I(i) = 43 (57.3%) I(ii) = 50 (62.5%) C = 80 (80%) 16 weeks: N = 169 (66%) I(i) = 46 (61.3%) I(ii) = 43 (53.8%) C = 80 (80%)	Not reported. Mean logons: I(i) = 5.9 (SD = 6.2; range 1-33) I(ii) = 5.6 (SD = 5.8; range = 1-27)	Baseline depressive symptoms (CES-D) and age lower in those who completed at least one follow-up questionnaire (*P* < .05). Gender not a predictor. Control participants more likely to complete a follow-up assessment. Mean logons did not differ between postcard & telephone reminder conditions (p > .05).	No	ITT random effect regression analyses – REML
Patten 2003 [29] (4 content modules)	N = 786 I = 420 C = 366	1 month: I = 418 (99.5%) C = 363 (99.2%) 2 months: I = 412 (98.1%) C = 361 (98.6%) 3 months: I = 406 (96.7%) C = 358 (97.8%)	Not reported. Mean duration signed-on = 50 min	None reported.	No	Completers
Spek et al 2007 [30] Spek et al 2008 [31] (12 month follow-up) I(i) = 8 modules I(ii) = 10 sessions	N = 301 I(i) = 102 I(ii) = 99 C = 100 *I(i) = Internet CBTI(ii) = Group CBT*	Post-treatment: N = 181 (60.1%) I(i) = 67 (65.7%) I(ii) = 56 (56.6%) C = 58 (58%) 12 months: N = 190 (63.1%) I(i) = 58 (56.8%) I(ii) = 66 (66.6%) C = 66 (66%)	Mean modules/sessions completed: I(i) = 5.5 out of 8 (78.1%) I(ii) = 9.1 out of 10 (98.3%) Completed whole course: I(i) = 48.3% I(ii) = 94.5%	Less treatment completion in Internet intervention group.	No formal measure described. Main reason reported: lack of time.	ITT MI
Warmerdam et al 2008 [32] (I(i) = 9 lessons, I(ii) = 5 lessons)	N = 263 I(i) = 88 I(ii) = 88 C = 87 *I(i) = Cognitive Behavioral Therapy (CBT)I(ii)=Problem Solving Therapy (PST)*	5 weeks: N = 184 (69.9%) I(i) = 61 (69.3%) I(ii) = 52 (59.1%) C = 71 (81.6%) 8 weeks: N = 173 (65.8%) I(i) = 51 (57.9%) I(ii) = 51 (57.9%) C = 71 (81.6%) 12 weeks: N = 151 (57.4%) I(i) = 46 (52.2%) I(ii) = 42 (47.2%) C = 63 (72.4%)	Completed at least 1 module: I(i) = 80 (90.9%) I(ii) = 74 (84.1%) Completed at least 3-4 lessons: I(i)=63 (71.6%) I(ii) = 49 (55.7%) Completed whole course: I(i) = 34 (38.6%) I(ii) = 33 (37.5%)	Lower withdrawal in control group compared with both intervention groups. Participants who completed post-treatment measures more likely to be born in the Netherlands and older.	No formal measure described. Reported reasons: other treatment; feeling better; lack of time; and problems understanding the program.	ITT LLM using REML
**Depression, anxiety and stress**						
van Straten et al 2008 [33] (4 modules)	N = 213 I = 107 C = 106	Post-treatment: N = 177 (83.1%) I = 81 (76%) C = 96 (91%)	Completed 1 module = 97 (90.6%) Completed 2 modules = 79 (73.8%) Completed 3 modules = 70 (65.4%) Completed whole course = 59 (55.1%)	Post-treatment measure response rate higher among more educated participants and those without alcohol problems. Married participants more likely to complete the intervention.	No	ITT MI
**Generalised anxiety disorder**						
Kenardy et al 2003 [34] (6 modules) Kenardy et al 2006 [35] (6 month follow-up)	N = 83 I = 43 C = 40	Post-treatment N = 75 (90.4%) I = 37 (86%) C = 38 (95%) 6 months N = 42 (50.6%) I = 19 (44.2%) C = 23 (57.5%)	Average modules completed = 3.33 out of 7 (SD = 2.10). Mean logons = 7.76 (SD = 7.31). Mean access time = 90.37 minutes (SD = 111.29).	Baseline depressive symptoms (CES-D), anxiety sensitivity (ASI) lower among completers than dropouts. At 6 months: No differences between those who dropped out in this period and those who did not.	No formal measure described. Main reason reported: time constraints. At 6 months: No reasons for additional dropout between post-test and 6 months reported.	Completers. Excluded outlier (high post test results in the intervention group; n = 1).
**Panic disorder**						
Carlbring et al 2001 [36] (6 modules)	N = 41 I = not reported C = not reported	Post-treatment: N = 36 (87.9%) I = 4 dropouts C = 1 dropout	Completed all modules: 100% (excluding participants who dropped out)	None reported.	No formal measure described. Reported reasons: I = lack of time (n = 3); serious physical illness (n = 1). C = no reason given.	ITT LOCF
Carlbring et al 2006 [37] (10 modules)	N = 60 I = 30 C = 30	Post-treatment: N = 57 (95%) I = 28^d^ (93.3%) C = 29 (96.6%) 9 months: I = 26 (86.6%) C = not collected	Completed all modules = 24 (80%); Mean number of modules completed = 8.9 (SD = 2.6). One participant completed 0 modules.	None reported.	No formal measure described. Reported reason: shortage of time (n = 1).	ITT LOCF
Klein and Richards, 2001 [38]	N = 23 I = 11 C = 12	Post-treatment: N = 22 (95.7%) C = not reported I = not reported	Not reported.	None reported.	No	Completers
Klein et al 2006 [39] (6 modules)	N = 55 I(i) = 19 I(ii) = 18 C = 18 *I(i) = Online CBTI(ii) = Manualized CBT*	Post-treatment: N = 46 (83.6%) I(i) = 18 (94.7%) I(ii) = 15 (83.3%) C = 13 (72.2%)	Those lost to follow-up did not complete the intervention.	Condition did not affect attrition.	No formal measure described. Reported reasons: I(i) = bipolar disorder episode (n = 1). I(ii) = depressive episode ( n = 1); treatment perceived to be ineffective (n = 1); lack of motivation (n = 1). C = monitoring led to recurrence of ‘bad’ memories (n = 1); no reason given (n = 4).	ITT LOCF
Richards et al 2006 [40]	N = 32 I(i) = 12 I(ii) = 11 C = 9 *I(i) = Online CBTI(ii) = Online CBT + stress management*	Post-treatment: N = 27 (84.4%) I(i) = 10 (83.3%) I(ii) = 10 (90.9%) C = 7 (77.8%)	Not reported.	Completers frequency of emails I(i) = 15.3 (SD = 12.8) I(ii) = 11.6 (SD = 13.3)	No formal measure described. Reported reasons: I(i) = lack of motivation, episode of depression I(ii) = wish to commence SSRI C = no reason given.	ITT LOCF
**Social phobia**						
Andersson et al 2006 [41]	N = 64 I = 32 C = 32	Post-treatment: N = 62 (96.9%) I = 30 (93.8%) C = 32 (100%) 12 months: N = 49 (76.6%) I = 29 (90.6%) C = 20 (62.5%)	Completed all modules = 20 (62.5%) Mean modules completed = 7.5 (SD = 2.4)	None reported.	No formal measure described. Reported reason: lack of time.	ITT LOCF
Carlbring et al 2007 [42] (9 modules)	N = 57 C = 30 I = 30	Post-treatment: N = 55 (96.5%) C = 28 (93.3%) I = 28 (93.3%) 12 months: I = 27 (90%) C = Not collected	Completed whole course = 27 (93.1%) Completed 4 modules = 1 (3.4%) Completed 1 module = 1 (3.4%)	None reported.	No formal measure described. Reported reasons for dropout: I = began other therapy (n = 1); No computer access (n = 1) C = began other therapy (n = 1) Reasons for not completing treatment: lack of time	Analysis excluded two participants after randomization but included two partially treatment compliant participants and one participant who did not return post-survey using LOCF.
Titov et al 2008 [43] (6 modules)	N = 105 I = 50 C = 55	Post-treatment: N = 93 (88.6%) I = 44 (88%) C = 49 (89.1%)	39 (78%) completed whole course	None reported	No formal measure described. Reported reasons: lack of time and motivation (n = 2); exposure too anxiety provoking (n = 1); programme not helpful (n = 1); overseas holiday (n = 1); change in work or study commitments (n = 3); medical complications (n = 1); no reason (n = 2)	ITT LOCF
Titov et al 2008 [44]	N = 88 I = 43 C = 45	Post-treatment: N = 78 (88.6%) I = 38 (88.4%) C = 40 (88.8%)	33 (73.3%) completed whole course Mean modules completed: 5.5 out of 6	None reported.	No formal measure described. Reported reasons: programme not helpful ( n =1); symptoms improved significantly (n = 1)	ITT LOCF
**Post traumatic stress disorder**						
Hirai and Clum 2005 [45]	N = 36 I = 18 C = 18	Post-treatment: N = 27 (75%) I = 13 (72.2%) C = 14 (77.8%)	Not reported.	No demographic differences were found between completers and those who dropped out.	No	Completers
Knaevelsrud et al 2007 [46] (10 sessions)	N = 96 I = 49 C = 47	Post-treatment: N = 87 (90.6%) I = 41 (83.7%) C = 46 (97.9%) 3 months: I = 41 (83.7%) C = not assessed	Not reported	None reported	No formal measure described. Reported reasons include: technical problems (with network and computer) and emotional distress.	ITT LOCF
Lange et al 2001 [47]	N = 30 I = 15 C = 15	Post-treatment: N = 25 (83.3%) C = 12 (80%) I = 13 (86.7%)	Not reported.	Participants who dropped out showed lower baseline intrusion scores (Impact of Events scale).	No formal measure described. Reported reasons: No quiet place for writing; could not focus on one trauma; ceased studies; marked improvement so saw no value in continuing.	Completers
Lange et al 2003 [48]	N = 184 I = 122 C = 62	Post-treatment: N = 101 (54.9%) I = 69 (56.6%) C = 32 (51.6%) 6 weeks: I = 57 (46.7%) C = not collected	Completed treatment = 78 (63.9%)	Compliance with treatment higher for women, for older people, for those who lived with a partner, those less experienced with a computer. Education, time since trauma, amount disclosed about trauma, and psychological functioning did not predict adherence. Compliance with protocol was not predicted by any of the variables investigated.	Formal questionnaire administered. Reasons for dropout: Technical problems with computer (n = 18, 41%) Preference for face-to face contact (n = 13, 29.5%) Burden of writing about stressful events (n = 13, 29.5%) 6 weeks Reported reasons: failure to respond; sought ‘other treatment’; did not wish to wait.	Completers

Note: ITT = Intention to treat; NMAR = Not Missing at Random; MAR = Missing at Random; LOCF = Last Observation Carried Forward; REML = Restricted Maximum Likelihood Estimation; LLM = Linear Mixed Modelling; MI = Multiple Imputation using NORM procedure in statistical package R; CBT = cognitive behavioral therapy.

^a^Control involved an online discussion group.

^b^had received intervention at 3 months.

^c^The same website can be offered both as open access site directly to the community or as a Web-based intervention offered in a randomized controlled trial.

^d^In contrast to the authors of some papers, the dropout rate is calculated strictly using the number randomized as the denominator. Hence figures may differ from those reported by authors in some cases (e.g., Carlbringet al 2007 [42]).

### Rates of Dropout/Non-completion of Study Protocol

Completion of protocol rates for depression sites ranged from a low of 43% [23] to a high of 99% [29], with some trials indicating poorer retention after a longer follow-up [26]. All studies reported lower rates of completion in the experimental intervention group relative to the control with the exception of Spek et al [30]. The one GAD trial reported a 6-month follow-up retention of 44% in the experimental group [34]. Trials for PD reported high rates of retention—approximately 80 - 90% for the experimental group, but these were based on small numbers of participants, and rates of dropout were often not reported separately for experimental and control conditions. Rates of completion for the SP interventions were approximately 90% at 12-month follow-up. Rates for PTSD ranged from 87% at post-treatment [47] to 47% at 6 week follow-up [48].

### Adherence

Adherence data were reported using indications such as number of log ons, duration of Web exposure, number of modules or exercises completed, and number of postings on bulletin boards. Although rates varied considerably, adherence to the complete online treatment was approximately 50 - 70% for depression sites and 50% for the sole GAD intervention [34]. Rates of adherence to the PD interventions were reported as high as 80 - 100% [36,37]. The SP trials reported 70 - 90%, and one of the PTSD trials reported a rate of 64% [48].

### Predictors of Adherence

For depression, predictors found to be associated with increased adherence were lower baseline rates of depression, younger age, and poorer knowledge of psychological treatments. Education or quality of life, when measured, did not predict adherence. For GAD, lower symptom levels predicted better adherence. Data for PD trials were scant. One trial of a PTSD intervention reported higher adherence with treatment for women, older persons, those who lived with a partner, and those less experienced with a computer.

### Self-Reported Reason for Dropout

Only one study conducted a formal survey of the reasons for dropout [48]. However, the following were mentioned as reasons for dropout in the Internet intervention group or, where separate data were not provided, in the group of participants as a whole: time constraints [23,30,31,32,34,36,37,41,42,43], lack of motivation [39,40,43], technical or computer-access problems [42,46,48], depressive episode or physical illness [39,40], the lack of face-to-face contact [48], preference for taking medication [40], perceived lack of treatment effectiveness [39,43,44,47,48], improvement in condition [29,32,44,47], and burden of the program [23,48].

### Methods to Analyze Missing Data

For depression, four approaches to missing data were used: analysis of completers only [23, 29]; intention to treat (ITT) using last observation carried forward (LOCF) [6,24,25,26]; mixed models with maximum likelihood estimation (REML) [27,28,32], the latter being one of the best of the approaches and standard good practice [49]; and multiple imputation [30,31], also a recommended strategy [50]. For GAD, a completer analysis was conducted. Panic disorder studies reported two approaches: four studies used ITT with LOCF [36,37,39,40] and one analyzed completers alone [38]. All of the social phobia studies utilized LOCF. Three PTSD studies used completer analyses [45,47,48], and one study used ITT with LOCF [46].

## Discussion

### Findings

Relative to reported rates of dropout from open access sites, the present study found that the rates of attrition in RCTs were lower, ranging from a high loss of 50% to a low of 1% over various follow-up periods. Treatment adherence was relatively high, at over 50%. These rates are relatively similar to those in randomized controlled trials of non-Internet-based interventions for generalized anxiety disorder and depression, with a recent review suggesting attrition rates are about 15% on average for GAD, but the rate of dropout ranged from 0 - 50% [51]. Our findings suggest that there is nothing particularly non-adherent about an Internet intervention per se when delivered in the context of a randomized controlled trial. However, these findings confirm that dropout is much less dramatic than that associated with open access websites. As such, the findings clearly articulate the need to compare rates of adherence for open access interventions against appropriate benchmarks. In our view, the rates of adherence for open access websites should be compared to rates of adherence reported for traditional health services provided by practitioners face to face (Meichenbaum and Turk [52], page 25). Where reported, these data show that adherence rates are high in face-to-face treatment as well, with as many as 70% of patients missing by a third session, and hypothetical attrition curves indicating that almost 100% of users are non-adherent after 10 sessions. Stress, exercise, or smoking programs have estimated discontinuation rates of between 20 - 80% (see Turk and Meichenbaum [13], page 249), while anti-depressant medication is discontinued by approximately 40 - 80% (see Sabate [10], page 66).

The findings from our review of RCTs also need to be compared to other recent work on rates of adherence in Web treatments, including a recent review of barriers to the uptake of computerized cognitive behavior programs [53]. This review differs substantially from ours in that it used an integrative methodology (combining both qualitative and quantitative work), reported work up to July 2005 only, reviewed computer-based interventions in addition to Internet-based ones, and focused on CBT style interventions only. Its focus was also substantially different because it covered acceptability and satisfaction in addition to dropout. This review reported that a medium of 83% of participants completed the study (ie, did not dropout) and a medium of only 56% completed a course of the program in data from quantitative studies. Although these rates cannot be compared formally, they appear to be slightly lower than those of the present review. The medium dropout rate of interventions from the depression studies was 60%, while the adherence level ranged between 38 - 78%, depending on which outcome measure was used.

In our study, predictors of adherence were similar to previously identified factors [9], including disease severity, treatment length, and chronicity. Very few studies formally examined reasons for dropout, and it was noted that personal circumstances “played a major role, including travel (for those studies based around a clinic computer)” (see Waller and Gilbody [53], page 3). Most studies also failed to use appropriate statistical techniques to analyze missing data.

### Limitations of the Study

Measures of adherence to websites did vary across studies, and we acknowledge that the use of different methods (log ons vs modules completed, etc) will yield different measures of adherence, and that these measures will not necessarily correlate strongly. Website design will be another important factor in determining the type and richness of particular outcome measures. Further research is needed to determine whether a universal indicator of adherence using diverse measures could be developed. For example, a “percent” of adherence might be a useful approach. Both a strength and a limitation of the present study was its focus on anxiety and depression websites. Although beyond the scope of the present paper, there is a clear need to consolidate information about reliable predictors of adherence across other physical and mental disorders and diseases, and to identify both disease-specific and generic predictors.

The focus of this paper was to examine adherence in RCTs. It was not possible to compare directly the rates of adherence between open access services and trial-based Web interventions. To our knowledge the open access websites reported in the introduction are the only ones for which there is published data. With further publication of data for open access sites, it may be possible to undertake a formal review of predictors and rates. Once sufficient trials and evaluations have been conducted within open access sites and websites used as part of RCT trials, techniques to develop appropriate quantitative comparisons between efficacy and effectiveness studies could be systematically employed to compare these rates (see Hunsley and Mash [54]).

### Implications for Future Work

The findings from our review reinforce conclusions that have been drawn from traditional intervention research. Little is known about the specific component factors that improve adherence in health interventions. Research within this area is essentially atheoretical, and a coherent approach is required. Given the importance of adherence research, and the unique advantage of Web-based data collection for analyzing adherence, we suggest a potential research agenda to advance this area.

A first step requires the adoption of a theoretical approach to the understanding of dropout and adherence. The framework adopted by WHO [10] identifies five dimensions to pursue: health system factors, socioeconomic factors, therapy-related factors, condition-related factors, and patient-related factors. Historically, the emphasis has been on patient factors. For example, according to Davis and Addis, “What is needed are theories which link specific client characteristics and treatment processes onto attrition” [12] (page 347). However, there is now recognition that health systems factors seem critical. A substantial body of research on depression interventions in primary care emphasizes the importance of case management and continuity of care for efficacy and adherence [55]. These findings, together with the overall greater adherence rates achieved within the context of RCTs, point to the potential benefits of incorporating simple procedures such as monitoring and follow-up to increase adherence. This in turn suggests that attention to behavior theory/modification approaches may yield the greatest benefits for increasing adherence to open access websites. Information on the effectiveness of types, frequency, and size of rewards, as well as information on appropriate reinforcement schedules, is likely to be highly useful in developing comprehensive adherence programs. The lessons learned within research contexts for improving adherence to trial protocols [51] might be profitably employed in the design of better treatment delivery systems in community practice, although recommendations such as “if in doubt, screen out” are counter-productive to the aims of open access websites, which aim to reach individuals who are not yet committed to a treatment program. Research from the Internet intervention field already suggests that substantial gains might be achieved by using email tracking. Clarke et al [28], when comparing the outcomes of two trials of the Overcoming Depression on the Internet (ODIN) website, reported that reminders (both telephone and email) were likely to be the crucial factor in determining retention (and improvement). Studies of established Internet-based treatment programs indicate that high rates of adherence are indeed possible if case management and continuity of care principles are followed [56]. It is not yet known whether tailoring improves adherence in mental health, although this is often promoted as the cornerstone of health promotion campaigns, and it forms a rich area for potential investigation.

Other authors have pointed to the potential of a range of theoretical models other than those based on behavior theory/modification to inform programs for increasing adherence at an individual level, although these focus to a greater degree on person, rather than systems, factors. The health belief model [57] attempts to predict behavior on the basis of a person’s perception of the risks associated with a health condition, as well as beliefs about the costs, potential side effects/difficulties, and benefits of treatment. The protection motivation theory, the theory of reasoned action, the theory of planned behavior, the social-cognitive theory, and models based on self-efficacy [57] have also been proposed as theoretical models that might inform adherence practice.

A second area likely to advance our understanding of website adherence involves research into the methods by which technology engages users. Eysenbach [2] has cited specific factors such as usability and other technological factors that will have an impact on adherence. This research agenda potentially covers a range of areas: (1) the generic ways in which humans interact with the technology associated with the Internet (such as frequency of use); (2) the specific methods used by individuals to interact with intervention programs as realized on a website (such as skipping sections, completing online assessments); and (3) the means by which users engage preferentially with certain names or brands of website. The latter includes research into the nature of trust (see, for example, Corritore et al [58]). An example of (2), above, is research into the effectiveness of various forms of the presentation of multimedia (see, for example, Sun and Cheng [59]). The investigation of the ways in which humans interact with computers and the Internet (as in (1), above) is also a potentially interesting area for future research. Many users report forming attachments to their computers (an observation that can be confirmed by undertaking a quick search on Google with the phrase “I love my computer”), and a better understanding of the “computer therapeutic alliance” might well be justified.

Internet interventions have a number of unique features that may impact on adherence rates. These features include the ease with which the interventions can be accessed, the expectations of users, the level of contact with a health professional, and the presence of rewards or motivators. Although evidence is lacking, Internet delivery may increase adherence relative to face-to-face interventions for individual users who respond to the interactivity, tailoring, and online rewards associated with some websites. One hypothesis worthy of investigation is that Internet delivery creates a technology “alliance”, reflecting the attraction or attachment which develops between people and electronic gadgets and computers. Moreover, the hypothesis that websites attract people who prefer treatment delivered anonymously, prefer distal contact, or are housebound because of mental or physical disability requires testing.

A third issue that warrants further detailed investigation is the role of disease factors and their influence on treatment uptake and maintenance. The cognitive and emotional characteristics of individuals with depression (or anxiety) are likely to impact their choice of treatment, their treatment uptake, and their rates of adherence. As part of their condition, individuals with depression may believe that they do not deserve treatment or that their treatment is unlikely to be effective and, as a consequence, they may be more likely to drop out. Depressed individuals may simply not be able to face using a computer. Intervention programs that directly address “cognitive dysfunctional thoughts” about treatment outcomes may produce better adherence and outcomes.

This review also identified a number of methodological improvements that are needed to advance the area. Often terms such as adherence, compliance, attrition, and dropout are not operationalized and are used interchangeably. Many studies fail to measure adherence to treatment. Statistical approaches to handling missing data are limited. Use of mixed models with REML approaches to missingness, rather than the use of biased methods such as LOCF or limited methods such as completer analyses, are to be encouraged [49,50]. There may be a need for reanalysis of research trials which are already published.

Finally, it is appropriate to consider the implications of these findings for identifying ways of reducing the high attrition rates on open access websites. Based on the data from RCTs, it seems likely that adherence to open access sites might immediately be improved if users were to consent to the use of automated reminders and messages. It is likely that the use of automated reminders will be successful across a range of interventions, not just those directed at anxiety and depression. Research investigating the acceptability and effectiveness of such a tracking procedure for open access sites should be accorded a high level of priority.

## Abbreviations

CBT: cognitive behavioural therapy
GAD: generalized anxiety disorder
ITT: intention to treat
LLM: linear mixed modelling
LOCF: last observation carried forward
MAR: missing at random
MI: multiple imputation
ML: maximum likelihood methods
NMAR: not missing at random
PD: panic disorder
PTSD: post-traumatic stress disorder
RCT: randomized controlled trials
REML: restricted maximum likelihood estimation
SP: social phobia
WHO: World Health Organization
